# Efficient OCT Image Enhancement Based on Collaborative Shock Filtering

**DOI:** 10.1155/2018/7329548

**Published:** 2018-02-01

**Authors:** Guohua Liu, Ziyu Wang, Guoying Mu, Peijin Li

**Affiliations:** ^1^Department of Ophthalmology, Qilu Children's Hospital of Shandong University, Jinan 250022, China; ^2^Department of Radiology, Yidu Central Hospital of Weifang, Qingzhou 262500, China; ^3^Department of Ophthalmology, Shandong Provincial Hospital, Jinan 250021, China; ^4^School of Computer Science and Technology, Shandong University, Jinan 250101, China

## Abstract

Efficient enhancement of noisy optical coherence tomography (OCT) images is a key task for interpreting them correctly. In this paper, to better enhance details and layered structures of a human retina image, we propose a collaborative shock filtering for OCT image denoising and enhancement. Noisy OCT image is first denoised by a collaborative filtering method with new similarity measure, and then the denoised image is sharpened by a shock-type filtering for edge and detail enhancement. For dim OCT images, in order to improve image contrast for the detection of tiny lesions, a gamma transformation is first used to enhance the images within proper gray levels. The proposed method integrating image smoothing and sharpening simultaneously obtains better visual results in experiments.

## 1. Introduction

Optical coherence tomography (OCT) is an important emerging noninvasive technique in medical imaging, which offers high-speed tomographic imaging of human tissues [1, 2]. Layered structures of the human retina provided by OCT imaging are commonly detected and measured for diagnosis and prognostic evaluation [3]. Thus, medical OCT imaging has a variety of clinical applications and is widely used in the ophthalmology for vision-related diseases. Since OCT imaging takes advantage of low-coherence interferometry, it inevitably suffers from speckle noise, visible as dark and bright spots on the imaged objects [4], which severely degrades OCT image contrast and quality and makes it particularly challenging to identify fine structures and features. Therefore, it is necessary to remove the speckle noise and to enhance OCT images in order to interpret OCT images correctly.

There are many approaches to reduce the speckle noise of OCT images for enhancing their image quality [5, 6]. For example, the geometry-oriented calculus of variations and partial differential equations have been widely used in image processing [7, 8]: the total variation regularization [9] and the anisotropic diffusion [10] for image denoising and enhancement, the shock filters [11, 12] for image sharpening, and so forth. However, in the process of image denoising and enhancement, these classic geometrical regularization methods, based on operators in differential geometry such as gradient, divergence, and directional derivative, often tend to modify the image towards a piecewise constant function and blur fine features of the image, particularly the image's details and layered structures.

Other image enhancement methods include wavelet decomposition [13], local statistical [14], and self-similarity and sparsity-based methods [15–17]. These algorithms can reduce noise to a certain extent, but they also lead to the loss of image details and consequently the blur of important image features. Moreover, some algorithms are fairly time-consuming for them to enhance a noisy OCT image.

In 2005, Buades et al. proposed a nonlocal means (NLM) filter based on the self-similarity in the whole image, which can effectively preserve image details and textures [18, 19]. The nonlocal means filter produces impressive results in denoising textured patterns. A more complicated neighborhood filter based on the self-similarity of image blocks was introduced by Dabov et al. [20], which is actually also a weighted average algorithm, just after a transform-domain collaborative filtering. This algorithm achieves better denoising performance in terms of peak signal-to-noise ratio [21].

In this paper, to better preserve or enhance image details and layered structures, we propose an effective enhancement algorithm for OCT image denoising and enhancement based on collaborative shock filtering. The basic idea is that noisy OCT image is first denoised with a collaborative filtering of speckle noise following a gamma distribution, and then the denoised image is sharpened by a shock-type filtering for edge and detail enhancement. For dim OCT image, in order to improve the contrast of the retinal image for the detection of tiny lesions, a gamma transformation [22] is first used to enhance the image within proper gray levels.

Image smoothing and sharpening are two opposite operations in image processing. Generally speaking, image smoothing is to eliminate unnecessary and false discontinuous features (such as noise), while image sharpening is to produce or enhance some discontinuous features (such as edges and details) in proper positions of the image. In many cases, image denoising methods often inevitably blur image edges and details even if these methods are designed elaborately. For example, see following denoising experiments on noisy images. Thus, both image smoothing and sharpening are needed simultaneously even if only in an image denoising task. One will see that the methods integrating the above two operations simultaneously obtain better visual results in experiments [23].

The rest of this paper is organized as follows. In Section 2, we briefly review some classic works: shock filters and self-similarity filtering. Then, a collaborative shock-filtering algorithm integrating these classic methods is proposed in Section 3. Experiments on test images are described in Section 4. Finally, conclusions are drawn out in Section 5.

## 2. Shock Filters and Self-Similarity Filtering

In this section, we review some classic methods in image denoising and enhancement.

### 2.1. Shock Filter

In [24], some special ideas and techniques developed in numerical solutions of nonlinear hyperbolic equations were applied to feature-oriented image enhancement for the first time. Osher and Rudin introduced a novel image sharpening technique called the shock filter (SF) [11], which is based on a modification of the nonlinear Burgers' equation and simulates the shock wave calculation in the computational fluid mechanics [25]. Different from the nonlinear parabolic equation of diffusion-type process, they proposed a hyperbolic one:

(1) $$\begin{array}{c} \frac{\partial u}{\partial t}=-\mathrm{sign}\left(u_{NN}\right)\left|\nabla u\right|, \end{array}$$

where *u* is the observed noisy image, sign is a sign function, and *u*_*NN*_ is the second directional derivative of the image along a local normal direction to the isophote line. It detects an image edge using the zero crossing of *u*_*NN*_, where a shock is formed at the speed of ∣*∇u*∣.

Considering the image noise in the estimation of edges, Alvarez and Mazorra added a smoothing kernel and coupled the anisotropic diffusion with the shock filter (ADSF) [12] for noise elimination and edge sharpening:

(2) $$\begin{array}{c} \frac{\partial u}{\partial t}=-\mathrm{sign}\left(G_{\sigma }*u_{NN}\right)\;|\;\nabla u\;|\;+{cu}_{TT}, \end{array}$$

where *G*_*σ*_ is a Gaussian kernel with standard deviation *σ*, *u*_*TT*_ is the second directional derivative of the image along a local tangent direction, and *c* is a constant to balance the anisotropic diffusion and the shock filter.

### 2.2. Self-Similarity Filtering

Another effective image denoising technique is neighborhood filters [8], which consider self-similarity between two pixels or blocks of the image both in spatial location and in gray level. Buades et al. proposed the following nonlocal means (NLM) filter [18, 19]:

(3) $$\begin{array}{c} {NL}_{h}u\left(x\right)=\frac{1}{c\left(x\right)}\int_{\Omega }\exp\left(-\frac{\left(G_{\sigma }*{\left(u\left(x+\cdot \right)-u\left(y+\cdot \right)\right)}^{2}\right)\left(0\right)}{h^{2}}\right)u\left(y\right)dy, \end{array}$$

where *c*(*x*) = ∫_Ω_exp(−(*G*_*σ*_∗(*u*(*x* + ·) − *u*(*y* + ·))^2^)(0)/*h* ^2^)*dy* is a normalization factor, and *h* is a filtering parameter related to noise level, and {*x*, *y* ∈ Ω ⊂ **R** ^2^} are 2D spatial coordinates. The nonlocal means filter gives better results in denoising textured patterns.

A more complicated neighborhood filter called the Block-matching and 3D filtering (BM3D) algorithm was introduced by Dabov et al. [20], which has three main steps: grouping of similar image blocks, 3D collaborative filtering of these blocks in the spectrum domain, and aggregation of all local estimates. This algorithm includes basic and final estimations using the following weighted average:

(4) $$\begin{array}{c} \hat{u}\left(x\right)=\frac{\sum_{R}\sum_{S}w_{R,S}{\hat{U}}_{R,S}\left(x\right)}{\sum_{R}\sum_{S}w_{R,S}\chi_{S}\left(x\right)},\;x\in \Omega , \end{array}$$

where *R* and *S* denote the reference block and the similar block, respectively, *w* is the weight of the corresponding block, $\hat{U}\left(x\right)$ is the local block-wise estimate, and *χ*(*x*) is a characteristic function. As they claim, this algorithm is currently one of best denoising methods in terms of peak signal-to-noise ratio [20, 21].

Finally, as one will see, the neighborhood filters are mainly used for image denoising, while the shock filters can be used for both image denoising and enhancement simultaneously, because of their inherent local backward diffusion [25].

## 3. Collaborative Shock Filtering

The main information of an image is encoded in its edges, details, and structures. These components need to be processed in special ways to enhance the image as well as possible.

In order to better preserve or enhance image details and layered structures, we propose an efficient collaborative shock-filtering (CSF) algorithm for image denoising and enhancement, which fuses different advantages of previously mentioned classic methods: powerful capabilities of the self-similarity filtering in image edge denoising and the shock filters in edge sharpening.

**Figure 1:**
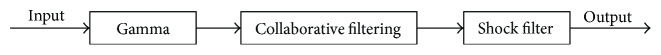
A flowchart of the proposed collaborative shock-filtering algorithm. A noisy OCT image is enhanced through three steps in sequence: gamma manipulation (gamma), collaborative filtering, and shock filter, respectively.

To be specific, as illustrated in Figure 1, for a given degraded OCT image, it is first denoised with a collaborative filtering method; then, the denoised image is sharpened by a shock-type filtering for edge and detail enhancement [11]. For dim OCT images, in order to improve the contrast of the retinal image for the detection of tiny lesions, a gamma transformation [22] is first used to enhance the image within proper gray levels.

Different from the BM3D algorithm for Gaussian noise removal [20], the block similarity measure is elaborated in the proposed collaborative filtering of speckle noise following a gamma distribution [26]. Based on a generalized likelihood ratio, the similarity criteria is defined as follows [27]:

(5) $$\begin{array}{c} S\left(A_{i},A_{j}\right)=\frac{mean\left(A_{i}⊙A_{j}\right)}{mean\left(\left(A_{i}+A_{j}\right)⊙\left(A_{i}+A_{j}\right)\right)}. \end{array}$$

Here, ⊙ is the Hadamard product of two image matrixes (blocks) and mean(·) is the function taking the mean of all elements of matrices. The larger value of *S* implies that image block *A*_*i*_ is more similar to *A*_*j*_.

## 4. Experimental Results

In this section, we perform a lot of enhancement experiments to verify our method on various degraded OCT images. For the above methods, numerical schemes presented in original works are used.

All methods are implemented using the MATLAB programming on OCT images with grayscale ranging from 0 to 1. In the comparison of different methods, parameters in each method are selected such that optimal visual results are obtained. Through three sequential processings of the gamma manipulation (*γ* = 0.6), the collaborative filtering (*σ* = 50) and the shock filter (Δ*t* = 0.05, *n* = 10), where *n* and Δ*t* denote the iteration times and the temporal step size, tiny details and layered structures are shown clearly due to image contrast improvement and noise removal. Moreover, our method produces fewer overshoot artifacts while avoiding noise magnification.

**Figure 2:**
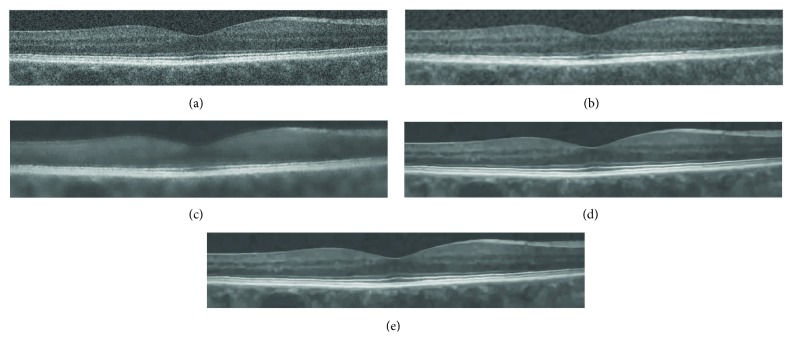
Enhancement of noisy OCT image: (a–e) original image, ADSF, NLM, BM3D, and the proposed collaborative shock filtering, respectively.

**Figure 3:**
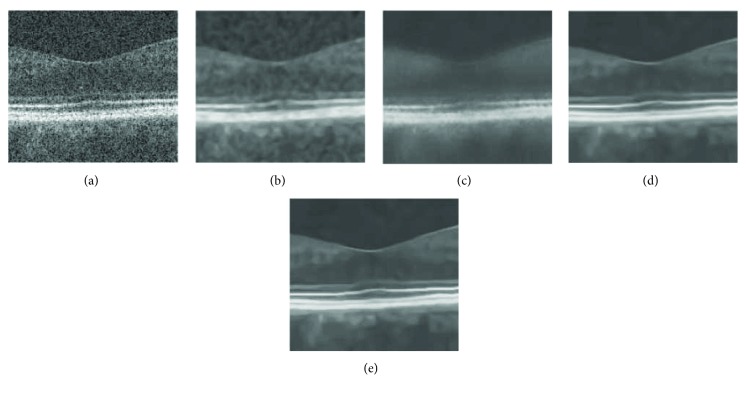
Zoomed parts in Figure 2: (a–e) original image, ADSF, NLM, BM3D, and the proposed collaborative shock filtering, respectively.

In Figure 2, a severely noisy OCT image of size 183 × 894 is enhanced without image gray adjustment (gamma transformation). Its better effects with clearer layered structures and effective noise removal can be observed more clearly in zoomed parts of results in Figure 3, compared with other methods such as ADSF, NLM, and BM3D. The ADSF method does not efficiently remove speckle noise and produces blurry edges and layered structures with some annoying artifacts in flat regions. The NLM method produces very blurred layered structures with broken nonsmooth linear edges. The BM3D method produces better enhancement results with a few slightly blurry edges and layered structures. Blurry and nonsmooth-layered structures are bad for one to measure the thickness of layered structures to predict and evaluate related disorders in ophthalmology.

**Figure 4:**
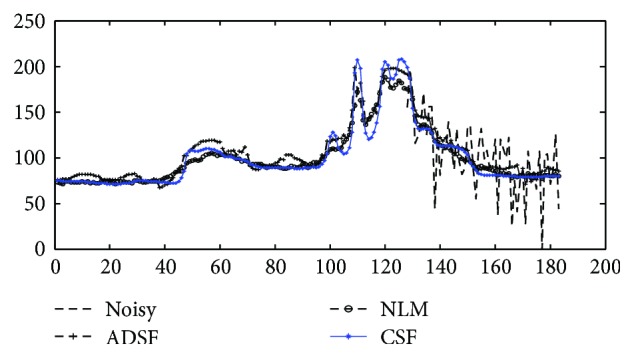
Enhancement of noisy OCT image: comparison of profiles (500th column) of original and enhanced images by ADSF, NLM, and the proposed collaborative shock filtering (CSF), respectively.

**Figure 5:**
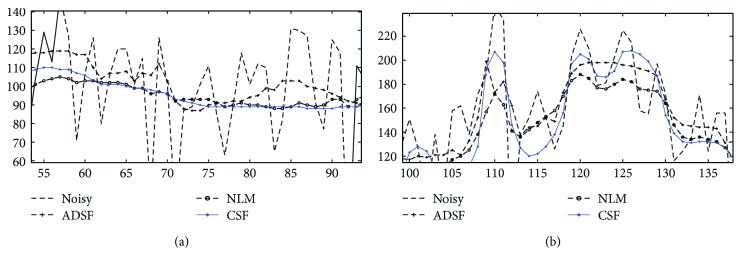
Enhancement of noisy OCT image: comparison of profiles of different regions of original and enhanced images by ADSF, NLM, and the proposed collaborative shock filtering (CSF): (a) flat region (line 55–90, 500th column) and (b) edge region (line 105–135, 500th column), respectively.

In order to observe enhancement effects by these methods more clearly, local profiles (500th column) of different results are shown in Figure 4. One can see that, compared with related methods, the proposed method removes speckle noise more effectively preserving layered structures without producing annoying artifacts, providing a chance to detect and evaluate retinal lesions faithfully by image enhancement. In Figure 5, local profiles of a flat region (line 55–90, 500th column) and an edge region (line 105–135, 500th column) of different results are shown. Superior denoising and edge sharpening by the proposed CSF method can be clearly observed: sharper edges with a smaller edge width are helpful for an accurate measure of layered structures.

**Figure 6:**
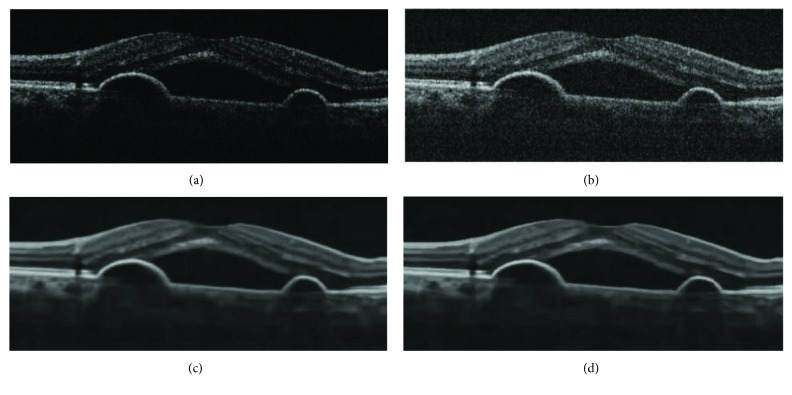
Enhancement of dim noisy OCT image: (a–d) original image, gamma transformation, BM3D, and the proposed collaborative shock filtering, respectively.

In Figure 6, a very dim and noisy OCT image of size 194 × 477 is enhanced with image gray adjustment. Clearer layered structures can be easily observed with effective noise removal.

**Figure 7:**
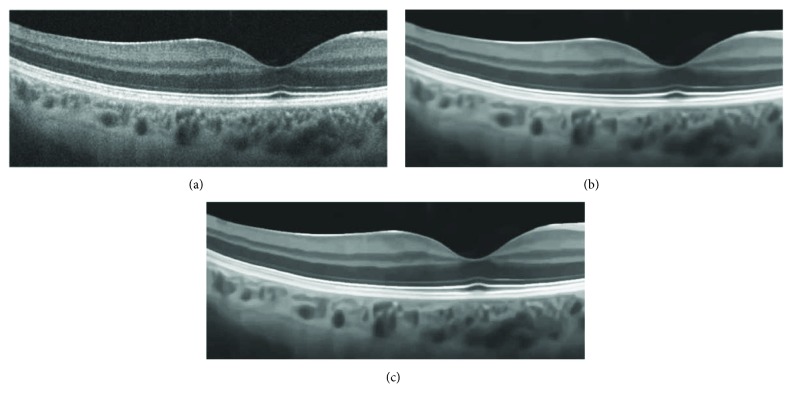
Enhancement of noisy OCT image: (a–c): original image, BM3D, and proposed collaborative shock filtering, respectively.

**Figure 8 fig8:**
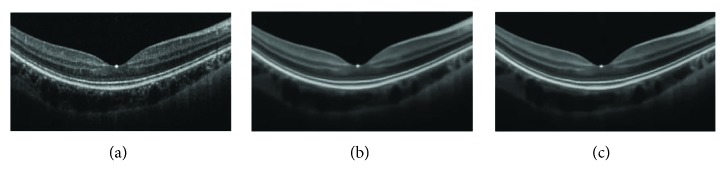
Enhancement of dim noisy OCT image (a–c) original image, BM3D, and the proposed collaborative shock filtering, respectively.

**Figure 9:**
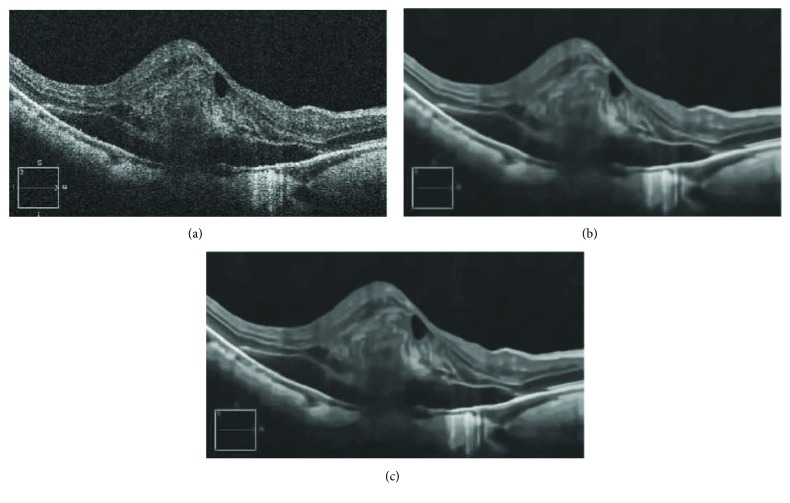
Enhancement of dim noisy OCT image: (a–c): original image, BM3D, and the proposed collaborative shock filtering, respectively.

In Figures 7 [Fig fig8]–9, three much noisy OCT images of sizes (245 × 586, 187 × 341, and 250 × 453) are enhanced without image gray adjustment. Clearer layered structures can be easily observed with effective noise removal.

**Figure 10:**
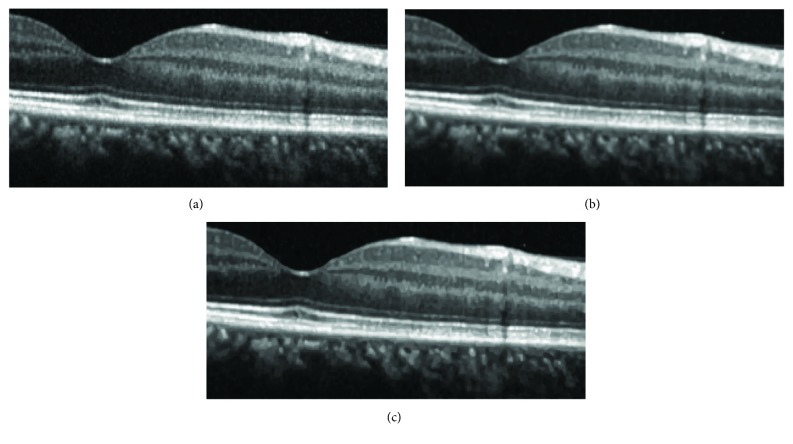
Enhancement of blurred noisy OCT image: (a–c) original image, BM3D, the and proposed collaborative shock filtering, respectively.

In Figure 10, a blurred noisy OCT image of size 594 × 1263 is enhanced without image gray adjustment. In this case, more sharpening with the shock filter (Δ*t* = 0.1, *n* = 10) is used to ensure clearer layered structures and effective noise removal.

**Figure 11:**
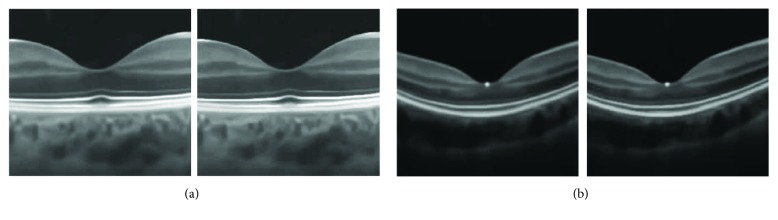
Enhancement of noisy OCT images: (a) results by BM3D and (b) results by the proposed collaborative shock filtering, respectively.

**Figure 12:**
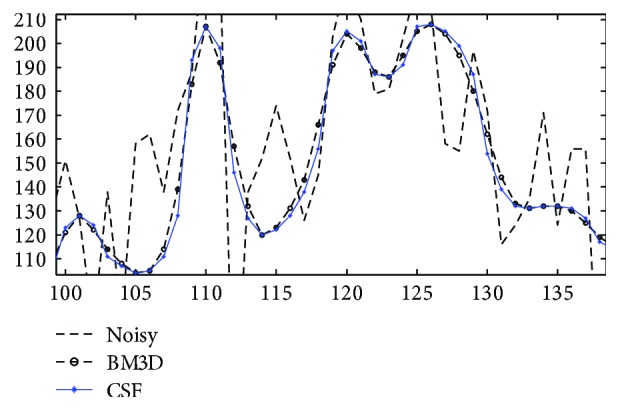
Enhancement of noisy OCT image (first test image in Figure 2): comparison of profiles (line 105–135, 500th column) of enhanced images by BM3D and the proposed collaborative shock filtering (CSF), respectively.

Finally, we compare the proposed CSF method with the BM3D method for edge sharpening in OCT image enhancement. In Figure 11, typical results of previous experiments by two methods are shown to carefully observe the enhancement of layered structures in OCT images. Obviously, the CSF method produces sharper edges and clearer layered structures around the retinal fovea. In Figure 12, moreover, it is clearer for one to observe profiles of an edge region (line 105–135, 500th column) in enhancing the first test image in Figure 2: the proposed CSF method produces sharper edges with smaller edge width, which is consistent with the visual observation.

## 5. Conclusions and Future Work

To better enhance image details and layered structures of noisy optical coherence tomography (OCT) image, we propose a collaborative shock filtering for OCT image denoising and enhancement. Noisy OCT image is first denoised with a collaborative filtering method based on a new similarity measure, followed by a sharpening step by a shock-type filtering for edge and detail enhancement. Finally, in order to improve the contrast of dim OCT image, a gamma transformation is used to enhance the image within proper gray levels. Simultaneously, integrating the image smoothing and sharpening of the proposed method obtains better visual results in image enhancement experiments.

In future work, we will develop robust measurement tools for quantitative analysis of vision-related diseases based on this work.
